# Supplementary material for: Positively selected modifications in the pore of TbAQP2 allow pentamidine to enter Trypanosoma brucei
Source: eLife. 2020 Aug 11;9:e56416. doi: 10.7554/eLife.56416 (PMC7473772; doi:10.7554/eLife.56416)
Supplement: Supplementary file 2. — Mutated nucleotides and codons are indicated. [file elife-56416-supp2.docx]

| Reagent type (species) or resource | Designation | Gene sequence (altered base(s) highlighted): Amino acid codon (underlined) | Codon position:  Start (5') - End (3') |
| --- | --- | --- | --- |
| gene (*Trypanosoma brucei*) | TbAQP2W.T  (Tb927.10.14170) | ATGCAGAGCCAACCAGACAATGTGGCGTATCCCATGGAGCTACAAGCGGTTAATAAGGATGGAACAGTGGAGGTCCGTGTTCAGGGAAACGTTGACAACAGTAGCAATGAGCGGTGGGATGCAGATGTACAAAAACATGAGGTGGCGGAGGCTCAAGAGAAACCCGTGGGAGGCATCAACTTTTGGGCACCACGGGAACTGCGGCTAAATTACCGCGACTACGTGGCTGAATTTCTGGGAAACTTCGTCCTCATATATATCGCTAAGGGCGCGGTTATCACCTCACTACTTGTTCCAGATTTTGGGCTTCTCGGTCTTACGATTGGTATTGGTGTGGCTGTCACGATGGCTCTGTATGTTTCACTGGGCATCTCCGGTGGCCATCTCAACTCTGCCGTCACCGTTGGCAATGCGGTTTTCGGTGATTTCCCTTGGAGAAAAGTCCCCGGCTACATCGCGGCGCAGATGCTCGGCACTTTCCTTGGTGCTGCCTGCGCTTACGGAGTGTTTGCTGATCTCCTGAAGGCGCATGGTGGTGGTGAGTTGATTGCCTTCGGTGAAAAGGGGATTGCGTGGGTGTTTGCCATGTACCCCGCGGAAGGAAATGGTATATTTTATCCAATTTTTGCTGAACTCATTTCCACCGCAGTGCTACTGCTCTGTGTCTGCGGTATCTTTGACCCCAATAACTCTCCTGCCAAGGGGTACGAAACGGTAGCTATTGGTGCTCTTGTCTTCGTCATGGTCAACAACTTCGGCTTAGCGTCTCCCCTTGCGATGAATCCCTCACTTGATTTCGGTCCCAGGGTCTTCGGTGCGATCCTTCTCGGGGGGGAAGTTTTTTCACATGCAAATTATTATTTCTGGGTTCCACTAGTTGTTCCATTCTTTGGAGCTATCCTTGGACTTTTTCTGTACAAATATTTTCTTCCACACTAA |  |
| gene (*Trypanosoma brucei*) | TbAQP2^S131P/S263A^ | ATGCAGAGCCAACCAGACAATGTGGCGTATCCCATGGAGCTACAAGCGGTTAATAAGGATGGAACAGTGGAGGTCCGTGTTCAGGGAAACGTTGACAACAGTAGCAATGAGCGGTGGGATGCAGATGTACAAAAACATGAGGTGGCGGAGGCTCAAGAGAAACCCGTGGGAGGCATCAACTTTTGGGCACCACGGGAACTGCGGCTAAATTACCGCGACTACGTGGCTGAATTTCTGGGAAACTTCGTCCTCATATATATCGCTAAGGGCGCGGTTATCACCTCACTACTTGTTCCAGATTTTGGGCTTCTCGGTCTTACGATTGGTATTGGTGTGGCTGTCACGATGGCTCTGTATGTTTCACTGGGCATCTCCGGTGGCCATCTCAACCCTGCCGTCACCGTTGGCAATGCGGTTTTCGGTGATTTCCCTTGGAGAAAAGTCCCCGGCTACATCGCGGCGCAGATGCTCGGCACTTTCCTTGGTGCTGCCTGCGCTTACGGAGTGTTTGCTGATCTCCTGAAGGCGCATGGTGGTGGTGAGTTGATTGCCTTCGGTGAAAAGGGGATTGCGGGGGTGTTTGCCATGTACCCCGCGGAAGGAAATGGTATATTTTATCCAATTTTTGCTGAACTCATTTCCACCGCAGTGCTACTGCTCTGTGTCTGCGGTATCTTTGACCCCAATAACTCTCCTGCCAAGGGGTACGAAACGGTAGCTATTGGTGCTCTTGTCTTCGTCATGGTCAACAACTTCGGCTTAGCGTCTCCCCTTGCGATGAATCCCGCACTTGATTTCGGTCCCAGGGTCTTCGGTGCGATCCTTCTCGGGGGGGAAGTTTTTTCACATGCAAATTATTATTTCTGGGTTCCACTAGTTGTTCCATTCTTTGGAGCTATCCTTGGACTTTTTCTGTACAAATATTTTCTTCCACACTAA | CCT: 391-393  GCA: 787-789 |
| gene (*Trypanosoma brucei*) | TbAQP2^L258Y^ | ATGCAGAGCCAACCAGACAATGTGGCGTATCCCATGGAGCTACAAGCGGTTAATAAGGATGGAACAGTGGAGGTCCGTGTTCAGGGAAACGTTGACAACAGTAGCAATGAGCGGTGGGATGCAGATGTACAAAAACATGAGGTGGCGGAGGCTCAAGAGAAACCCGTGGGAGGCATCAACTTTTGGGCACCACGGGAACTGCGGCTAAATTACCGCGACTACGTGGCTGAATTTCTGGGAAACTTCGTCCTCATATATATCGCTAAGGGCGCGGTTATCACCTCACTACTTGTTCCAGATTTTGGGCTTCTCGGTCTTACGATTGGTATTGGTGTGGCTGTCACGATGGCTCTGTATGTTTCACTGGGCATCTCCGGTGGCCATCTCAACTCTGCCGTCACCGTTGGCAATGCGGTTTTCGGTGATTTCCCTTGGAGAAAAGTCCCCGGCTACATCGCGGCGCAGATGCTCGGCACTTTCCTTGGTGCTGCCTGCGCTTACGGAGTGTTTGCTGATCTCCTGAAGGCGCATGGTGGTGGTGAGTTGATTGCCTTCGGTGAAAAGGGGATTGCGGGGGTGTTTGCCATGTACCCCGCGGAAGGAAATGGTATATTTTATCCAATTTTTGCTGAACTCATTTCCACCGCAGTGCTACTGCTCTGTGTCTGCGGTATCTTTGACCCCAATAACTCTCCTGCCAAGGGGTACGAAACGGTAGCTATTGGTGCTCTTGTCTTCGTCATGGTCAACAACTTCGGCTTAGCGTCTCCCTATGCGATGAATCCCTCACTTGATTTCGGTCCCAGGGTCTTCGGTGCGATCCTTCTCGGGGGGGAAGTTTTTTCACATGCAAATTATTATTTCTGGGTTCCACTAGTTGTTCCATTCTTTGGAGCTATCCTTGGACTTTTTCTGTACAAATATTTTCTTCCACACTAA | TAT: 772-774 |
| gene (*Trypanosoma brucei*) | TbAQP2I^110W^ | ATGCAGAGCCAACCAGACAATGTGGCGTATCCCATGGAGCTACAAGCGGTTAATAAGGATGGAACAGTGGAGGTCCGTGTTCAGGGAAACGTTGACAACAGTAGCAATGAGCGGTGGGATGCAGATGTACAAAAACATGAGGTGGCGGAGGCTCAAGAGAAACCCGTGGGAGGCATCAACTTTTGGGCACCACGGGAACTGCGGCTAAATTACCGCGACTACGTGGCTGAATTTCTGGGAAACTTCGTCCTCATATATATCGCTAAGGGCGCGGTTATCACCTCACTACTTGTTCCAGATTTTGGGCTTCTCGGTCTTACGATTGGTTGGGGTGTGGCTGTCACGATGGCTCTGTATGTTTCACTGGGCATCTCCGGTGGCCATCTCAACTCTGCCGTCACCGTTGGCAATGCGGTTTTCGGTGATTTCCCTTGGAGAAAAGTCCCCGGCTACATCGCGGCGCAGATGCTCGGCACTTTCCTTGGTGCTGCCTGCGCTTACGGAGTGTTTGCTGATCTCCTGAAGGCGCATGGTGGTGGTGAGTTGATTGCCTTCGGTGAAAAGGGGATTGCGGGGGTGTTTGCCATGTACCCCGCGGAAGGAAATGGTATATTTTATCCAATTTTTGCTGAACTCATTTCCACCGCAGTGCTACTGCTCTGTGTCTGCGGTATCTTTGACCCCAATAACTCTCCTGCCAAGGGGTACGAAACGGTAGCTATTGGTGCTCTTGTCTTCGTCATGGTCAACAACTTCGGCTTAGCGTCTCCCCTTGCGATGAATCCCTCACTTGATTTCGGTCCCAGGGTCTTCGGTGCGATCCTTCTCGGGGGGGAAGTTTTTTCACATGCAAATTATTATTTCTGGGTTCCACTAGTTGTTCCATTCTTTGGAGCTATCCTTGGACTTTTTCTGTACAAATATTTTCTTCCACACTAA | TGG:328-330 |
| gene (*Trypanosoma brucei*) | TbAQP2^L264R^ | ATGCAGAGCCAACCAGACAATGTGGCGTATCCCATGGAGCTACAAGCGGTTAATAAGGATGGAACAGTGGAGGTCCGTGTTCAGGGAAACGTTGACAACAGTAGCAATGAGCGGTGGGATGCAGATGTACAAAAACATGAGGTGGCGGAGGCTCAAGAGAAACCCGTGGGAGGCATCAACTTTTGGGCACCACGGGAACTGCGGCTAAATTACCGCGACTACGTGGCTGAATTTCTGGGAAACTTCGTCCTCATATATATCGCTAAGGGCGCGGTTATCACCTCACTACTTGTTCCAGATTTTGGGCTTCTCGGTCTTACGATTGGTATTGGTGTGGCTGTCACGATGGCTCTGTATGTTTCACTGGGCATCTCCGGTGGCCATCTCAACTCTGCCGTCACCGTTGGCAATGCGGTTTTCGGTGATTTCCCTTGGAGAAAAGTCCCCGGCTACATCGCGGCGCAGATGCTCGGCACTTTCCTTGGTGCTGCCTGCGCTTACGGAGTGTTTGCTGATCTCCTGAAGGCGCATGGTGGTGGTGAGTTGATTGCCTTCGGTGAAAAGGGGATTGCGGGGGTGTTTGCCATGTACCCCGCGGAAGGAAATGGTATATTTTATCCAATTTTTGCTGAACTCATTTCCACCGCAGTGCTACTGCTCTGTGTCTGCGGTATCTTTGACCCCAATAACTCTCCTGCCAAGGGGTACGAAACGGTAGCTATTGGTGCTCTTGTCTTCGTCATGGTCAACAACTTCGGCTTAGCGTCTCCCCTTGCGATGAATCCCTCACGTGATTTCGGTCCCAGGGTCTTCGGTGCGATCCTTCTCGGGGGGGAAGTTTTTTCACATGCAAATTATTATTTCTGGGTTCCACTAGTTGTTCCATTCTTTGGAGCTATCCTTGGACTTTTTCTGTACAAATATTTTCTTCCACACTAA | CGT: 790-792 |
| gene (*Trypanosoma brucei*) | TbAQP2^I110W/L264R^ | ATGCAGAGCCAACCAGACAATGTGGCGTATCCCATGGAGCTACAAGCGGTTAATAAGGATGGAACAGTGGAGGTCCGTGTTCAGGGAAACGTTGACAACAGTAGCAATGAGCGGTGGGATGCAGATGTACAAAAACATGAGGTGGCGGAGGCTCAAGAGAAACCCGTGGGAGGCATCAACTTTTGGGCACCACGGGAACTGCGGCTAAATTACCGCGACTACGTGGCTGAATTTCTGGGAAACTTCGTCCTCATATATATCGCTAAGGGCGCGGTTATCACCTCACTACTTGTTCCAGATTTTGGGCTTCTCGGTCTTACGATTGGTTGGGGTGTGGCTGTCACGATGGCTCTGTATGTTTCACTGGGCATCTCCGGTGGCCATCTCAACTCTGCCGTCACCGTTGGCAATGCGGTTTTCGGTGATTTCCCTTGGAGAAAAGTCCCCGGCTACATCGCGGCGCAGATGCTCGGCACTTTCCTTGGTGCTGCCTGCGCTTACGGAGTGTTTGCTGATCTCCTGAAGGCGCATGGTGGTGGTGAGTTGATTGCCTTCGGTGAAAAGGGGATTGCGGGGGTGTTTGCCATGTACCCCGCGGAAGGAAATGGTATATTTTATCCAATTTTTGCTGAACTCATTTCCACCGCAGTGCTACTGCTCTGTGTCTGCGGTATCTTTGACCCCAATAACTCTCCTGCCAAGGGGTACGAAACGGTAGCTATTGGTGCTCTTGTCTTCGTCATGGTCAACAACTTCGGCTTAGCGTCTCCCCTTGCGATGAATCCCTCACGTGATTTCGGTCCCAGGGTCTTCGGTGCGATCCTTCTCGGGGGGGAAGTTTTTTCACATGCAAATTATTATTTCTGGGTTCCACTAGTTGTTCCATTCTTTGGAGCTATCCTTGGACTTTTTCTGTACAAATATTTTCTTCCACACTAA | TGG: 328-330  CGT: 790-792 |
| gene (*Trypanosoma brucei*) | TbAQP2^I190T^ | ATGCAGAGCCAACCAGACAATGTGGCGTATCCCATGGAGCTACAAGCGGTTAATAAGGATGGAACAGTGGAGGTCCGTGTTCAGGGAAACGTTGACAACAGTAGCAATGAGCGGTGGGATGCAGATGTACAAAAACATGAGGTGGCGGAGGCTCAAGAGAAACCCGTGGGAGGCATCAACTTTTGGGCACCACGGGAACTGCGGCTAAATTACCGCGACTACGTGGCTGAATTTCTGGGAAACTTCGTCCTCATATATATCGCTAAGGGCGCGGTTATCACCTCACTACTTGTTCCAGATTTTGGGCTTCTCGGTCTTACGATTGGTATTGGTGTGGCTGTCACGATGGCTCTGTATGTTTCACTGGGCATCTCCGGTGGCCATCTCAACTCTGCCGTCACCGTTGGCAATGCGGTTTTCGGTGATTTCCCTTGGAGAAAAGTCCCCGGCTACATCGCGGCGCAGATGCTCGGCACTTTCCTTGGTGCTGCCTGCGCTTACGGAGTGTTTGCTGATCTCCTGAAGGCGCATGGTGGTGGTGAGTTGATTGCCTTCGGTGAAAAGGGGACTGCGGGGGTGTTTGCCATGTACCCCGCGGAAGGAAATGGTATATTTTATCCAATTTTTGCTGAACTCATTTCCACCGCAGTGCTACTGCTCTGTGTCTGCGGTATCTTTGACCCCAATAACTCTCCTGCCAAGGGGTACGAAACGGTAGCTATTGGTGCTCTTGTCTTCGTCATGGTCAACAACTTCGGCTTAGCGTCTCCCCTTGCGATGAATCCCTCACTTGATTTCGGTCCCAGGGTCTTCGGTGCGATCCTTCTCGGGGGGGAAGTTTTTTCACATGCAAATTATTATTTCTGGGTTCCACTAGTTGTTCCATTCTTTGGAGCTATCCTTGGACTTTTTCTGTACAAATATTTTCTTCCACACTAA | ACT: 568-570 |
| gene (*Trypanosoma brucei*) | TbAQP2^W192G^ | ATGCAGAGCCAACCAGACAATGTGGCGTATCCCATGGAGCTACAAGCGGTTAATAAGGATGGAACAGTGGAGGTCCGTGTTCAGGGAAACGTTGACAACAGTAGCAATGAGCGGTGGGATGCAGATGTACAAAAACATGAGGTGGCGGAGGCTCAAGAGAAACCCGTGGGAGGCATCAACTTTTGGGCACCACGGGAACTGCGGCTAAATTACCGCGACTACGTGGCTGAATTTCTGGGAAACTTCGTCCTCATATATATCGCTAAGGGCGCGGTTATCACCTCACTACTTGTTCCAGATTTTGGGCTTCTCGGTCTTACGATTGGTATTGGTGTGGCTGTCACGATGGCTCTGTATGTTTCACTGGGCATCTCCGGTGGCCATCTCAACTCTGCCGTCACCGTTGGCAATGCGGTTTTCGGTGATTTCCCTTGGAGAAAAGTCCCCGGCTACATCGCGGCGCAGATGCTCGGCACTTTCCTTGGTGCTGCCTGCGCTTACGGAGTGTTTGCTGATCTCCTGAAGGCGCATGGTGGTGGTGAGTTGATTGCCTTCGGTGAAAAGGGGATTGCGGGGGTGTTTGCCATGTACCCCGCGGAAGGAAATGGTATATTTTATCCAATTTTTGCTGAACTCATTTCCACCGCAGTGCTACTGCTCTGTGTCTGCGGTATCTTTGACCCCAATAACTCTCCTGCCAAGGGGTACGAAACGGTAGCTATTGGTGCTCTTGTCTTCGTCATGGTCAACAACTTCGGCTTAGCGTCTCCCCTTGCGATGAATCCCTCACTTGATTTCGGTCCCAGGGTCTTCGGTGCGATCCTTCTCGGGGGGGAAGTTTTTTCACATGCAAATTATTATTTCTGGGTTCCACTAGTTGTTCCATTCTTTGGAGCTATCCTTGGACTTTTTCTGTACAAATATTTTCTTCCACACTAA | GGG: 574-576 |
| gene (*Trypanosoma brucei*) | TbAQP2^I190T/W192G^ | ATGCAGAGCCAACCAGACAATGTGGCGTATCCCATGGAGCTACAAGCGGTTAATAAGGATGGAACAGTGGAGGTCCGTGTTCAGGGAAACGTTGACAACAGTAGCAATGAGCGGTGGGATGCAGATGTACAAAAACATGAGGTGGCGGAGGCTCAAGAGAAACCCGTGGGAGGCATCAACTTTTGGGCACCACGGGAACTGCGGCTAAATTACCGCGACTACGTGGCTGAATTTCTGGGAAACTTCGTCCTCATATATATCGCTAAGGGCGCGGTTATCACCTCACTACTTGTTCCAGATTTTGGGCTTCTCGGTCTTACGATTGGTATTGGTGTGGCTGTCACGATGGCTCTGTATGTTTCACTGGGCATCTCCGGTGGCCATCTCAACTCTGCCGTCACCGTTGGCAATGCGGTTTTCGGTGATTTCCCTTGGAGAAAAGTCCCCGGCTACATCGCGGCGCAGATGCTCGGCACTTTCCTTGGTGCTGCCTGCGCTTACGGAGTGTTTGCTGATCTCCTGAAGGCGCATGGTGGTGGTGAGTTGATTGCCTTCGGTGAAAAGGGGACTGCGGGGGTGTTTGCCATGTACCCCGCGGAAGGAAATGGTATATTTTATCCAATTTTTGCTGAACTCATTTCCACCGCAGTGCTACTGCTCTGTGTCTGCGGTATCTTTGACCCCAATAACTCTCCTGCCAAGGGGTACGAAACGGTAGCTATTGGTGCTCTTGTCTTCGTCATGGTCAACAACTTCGGCTTAGCGTCTCCCCTTGCGATGAATCCCTCACTTGATTTCGGTCCCAGGGTCTTCGGTGCGATCCTTCTCGGGGGGGAAGTTTTTTCACATGCAAATTATTATTTCTGGGTTCCACTAGTTGTTCCATTCTTTGGAGCTATCCTTGGACTTTTTCTGTACAAATATTTTCTTCCACACTAA | ACT: 568-570  GGG: 574-576 |
| gene (*Trypanosoma brucei*) | TbAQP2^L84W^ | ATGCAGAGCCAACCAGACAATGTGGCGTATCCCATGGAGCTACAAGCGGTTAATAAGGATGGAACAGTGGAGGTCCGTGTTCAGGGAAACGTTGACAACAGTAGCAATGAGCGGTGGGATGCAGATGTACAAAAACATGAGGTGGCGGAGGCTCAAGAGAAACCCGTGGGAGGCATCAACTTTTGGGCACCACGGGAACTGCGGCTAAATTACCGCGACTACGTGGCTGAATTTCTGGGAAACTTCGTCTGGATATATATCGCTAAGGGCGCGGTTATCACCTCACTACTTGTTCCAGATTTTGGGCTTCTCGGTCTTACGATTGGTATTGGTGTGGCTGTCACGATGGCTCTGTATGTTTCACTGGGCATCTCCGGTGGCCATCTCAACTCTGCCGTCACCGTTGGCAATGCGGTTTTCGGTGATTTCCCTTGGAGAAAAGTCCCCGGCTACATCGCGGCGCAGATGCTCGGCACTTTCCTTGGTGCTGCCTGCGCTTACGGAGTGTTTGCTGATCTCCTGAAGGCGCATGGTGGTGGTGAGTTGATTGCCTTCGGTGAAAAGGGGATTGCGGGGGTGTTTGCCATGTACCCCGCGGAAGGAAATGGTATATTTTATCCAATTTTTGCTGAACTCATTTCCACCGCAGTGCTACTGCTCTGTGTCTGCGGTATCTTTGACCCCAATAACTCTCCTGCCAAGGGGTACGAAACGGTAGCTATTGGTGCTCTTGTCTTCGTCATGGTCAACAACTTCGGCTTAGCGTCTCCCCTTGCGATGAATCCCTCACTTGATTTCGGTCCCAGGGTCTTCGGTGCGATCCTTCTCGGGGGGGAAGTTTTTTCACATGCAAATTATTATTTCTGGGTTCCACTAGTTGTTCCATTCTTTGGAGCTATCCTTGGACTTTTTCTGTACAAATATTTTCTTCCACACTAA | TGG: 250-252 |
| gene (*Trypanosoma brucei*) | TbAQP2^L84M^ | ATGCAGAGCCAACCAGACAATGTGGCGTATCCCATGGAGCTACAAGCGGTTAATAAGGATGGAACAGTGGAGGTCCGTGTTCAGGGAAACGTTGACAACAGTAGCAATGAGCGGTGGGATGCAGATGTACAAAAACATGAGGTGGCGGAGGCTCAAGAGAAACCCGTGGGAGGCATCAACTTTTGGGCACCACGGGAACTGCGGCTAAATTACCGCGACTACGTGGCTGAATTTCTGGGAAACTTCGTCATGATATATATCGCTAAGGGCGCGGTTATCACCTCACTACTTGTTCCAGATTTTGGGCTTCTCGGTCTTACGATTGGTATTGGTGTGGCTGTCACGATGGCTCTGTATGTTTCACTGGGCATCTCCGGTGGCCATCTCAACTCTGCCGTCACCGTTGGCAATGCGGTTTTCGGTGATTTCCCTTGGAGAAAAGTCCCCGGCTACATCGCGGCGCAGATGCTCGGCACTTTCCTTGGTGCTGCCTGCGCTTACGGAGTGTTTGCTGATCTCCTGAAGGCGCATGGTGGTGGTGAGTTGATTGCCTTCGGTGAAAAGGGGATTGCGGGGGTGTTTGCCATGTACCCCGCGGAAGGAAATGGTATATTTTATCCAATTTTTGCTGAACTCATTTCCACCGCAGTGCTACTGCTCTGTGTCTGCGGTATCTTTGACCCCAATAACTCTCCTGCCAAGGGGTACGAAACGGTAGCTATTGGTGCTCTTGTCTTCGTCATGGTCAACAACTTCGGCTTAGCGTCTCCCCTTGCGATGAATCCCTCACTTGATTTCGGTCCCAGGGTCTTCGGTGCGATCCTTCTCGGGGGGGAAGTTTTTTCACATGCAAATTATTATTTCTGGGTTCCACTAGTTGTTCCATTCTTTGGAGCTATCCTTGGACTTTTTCTGTACAAATATTTTCTTCCACACTAA | ATG: 250-252 |
| gene (*Trypanosoma brucei*) | TbAQP2^L118W^ | ATGCAGAGCCAACCAGACAATGTGGCGTATCCCATGGAGCTACAAGCGGTTAATAAGGATGGAACAGTGGAGGTCCGTGTTCAGGGAAACGTTGACAACAGTAGCAATGAGCGGTGGGATGCAGATGTACAAAAACATGAGGTGGCGGAGGCTCAAGAGAAACCCGTGGGAGGCATCAACTTTTGGGCACCACGGGAACTGCGGCTAAATTACCGCGACTACGTGGCTGAATTTCTGGGAAACTTCGTCCTCATATATATCGCTAAGGGCGCGGTTATCACCTCACTACTTGTTCCAGATTTTGGGCTTCTCGGTCTTACGATTGGTATTGGTGTGGCTGTCACGATGGCTTGGTATGTTTCACTGGGCATCTCCGGTGGCCATCTCAACTCTGCCGTCACCGTTGGCAATGCGGTTTTCGGTGATTTCCCTTGGAGAAAAGTCCCCGGCTACATCGCGGCGCAGATGCTCGGCACTTTCCTTGGTGCTGCCTGCGCTTACGGAGTGTTTGCTGATCTCCTGAAGGCGCATGGTGGTGGTGAGTTGATTGCCTTCGGTGAAAAGGGGATTGCGGGGGTGTTTGCCATGTACCCCGCGGAAGGAAATGGTATATTTTATCCAATTTTTGCTGAACTCATTTCCACCGCAGTGCTACTGCTCTGTGTCTGCGGTATCTTTGACCCCAATAACTCTCCTGCCAAGGGGTACGAAACGGTAGCTATTGGTGCTCTTGTCTTCGTCATGGTCAACAACTTCGGCTTAGCGTCTCCCCTTGCGATGAATCCCTCACTTGATTTCGGTCCCAGGGTCTTCGGTGCGATCCTTCTCGGGGGGGAAGTTTTTTCACATGCAAATTATTATTTCTGGGTTCCACTAGTTGTTCCATTCTTTGGAGCTATCCTTGGACTTTTTCTGTACAAATATTTTCTTCCACACTAA | TGG: 352-354 |
| gene (*Trypanosoma brucei*) | TbAQP2^L118M^ | ATGCAGAGCCAACCAGACAATGTGGCGTATCCCATGGAGCTACAAGCGGTTAATAAGGATGGAACAGTGGAGGTCCGTGTTCAGGGAAACGTTGACAACAGTAGCAATGAGCGGTGGGATGCAGATGTACAAAAACATGAGGTGGCGGAGGCTCAAGAGAAACCCGTGGGAGGCATCAACTTTTGGGCACCACGGGAACTGCGGCTAAATTACCGCGACTACGTGGCTGAATTTCTGGGAAACTTCGTCCTCATATATATCGCTAAGGGCGCGGTTATCACCTCACTACTTGTTCCAGATTTTGGGCTTCTCGGTCTTACGATTGGTATTGGTGTGGCTGTCACGATGGCTATGTATGTTTCACTGGGCATCTCCGGTGGCCATCTCAACTCTGCCGTCACCGTTGGCAATGCGGTTTTCGGTGATTTCCCTTGGAGAAAAGTCCCCGGCTACATCGCGGCGCAGATGCTCGGCACTTTCCTTGGTGCTGCCTGCGCTTACGGAGTGTTTGCTGATCTCCTGAAGGCGCATGGTGGTGGTGAGTTGATTGCCTTCGGTGAAAAGGGGATTGCGGGGGTGTTTGCCATGTACCCCGCGGAAGGAAATGGTATATTTTATCCAATTTTTGCTGAACTCATTTCCACCGCAGTGCTACTGCTCTGTGTCTGCGGTATCTTTGACCCCAATAACTCTCCTGCCAAGGGGTACGAAACGGTAGCTATTGGTGCTCTTGTCTTCGTCATGGTCAACAACTTCGGCTTAGCGTCTCCCCTTGCGATGAATCCCTCACTTGATTTCGGTCCCAGGGTCTTCGGTGCGATCCTTCTCGGGGGGGAAGTTTTTTCACATGCAAATTATTATTTCTGGGTTCCACTAGTTGTTCCATTCTTTGGAGCTATCCTTGGACTTTTTCTGTACAAATATTTTCTTCCACACTAA | ATG: 352-354 |
| gene (*Trypanosoma brucei*) | TbAQP2^L218W^ | ATGCAGAGCCAACCAGACAATGTGGCGTATCCCATGGAGCTACAAGCGGTTAATAAGGATGGAACAGTGGAGGTCCGTGTTCAGGGAAACGTTGACAACAGTAGCAATGAGCGGTGGGATGCAGATGTACAAAAACATGAGGTGGCGGAGGCTCAAGAGAAACCCGTGGGAGGCATCAACTTTTGGGCACCACGGGAACTGCGGCTAAATTACCGCGACTACGTGGCTGAATTTCTGGGAAACTTCGTCCTCATATATATCGCTAAGGGCGCGGTTATCACCTCACTACTTGTTCCAGATTTTGGGCTTCTCGGTCTTACGATTGGTATTGGTGTGGCTGTCACGATGGCTCTGTATGTTTCACTGGGCATCTCCGGTGGCCATCTCAACTCTGCCGTCACCGTTGGCAATGCGGTTTTCGGTGATTTCCCTTGGAGAAAAGTCCCCGGCTACATCGCGGCGCAGATGCTCGGCACTTTCCTTGGTGCTGCCTGCGCTTACGGAGTGTTTGCTGATCTCCTGAAGGCGCATGGTGGTGGTGAGTTGATTGCCTTCGGTGAAAAGGGGATTGCGGGGGTGTTTGCCATGTACCCCGCGGAAGGAAATGGTATATTTTATCCAATTTTTGCTGAACTCATTTCCACCGCAGTGTGGCTGCTCTGTGTCTGCGGTATCTTTGACCCCAATAACTCTCCTGCCAAGGGGTACGAAACGGTAGCTATTGGTGCTCTTGTCTTCGTCATGGTCAACAACTTCGGCTTAGCGTCTCCCCTTGCGATGAATCCCTCACTTGATTTCGGTCCCAGGGTCTTCGGTGCGATCCTTCTCGGGGGGGAAGTTTTTTCACATGCAAATTATTATTTCTGGGTTCCACTAGTTGTTCCATTCTTTGGAGCTATCCTTGGACTTTTTCTGTACAAATATTTTCTTCCACACTAA | TGG: 652-654 |
| gene (*Trypanosoma brucei*) | TbAQP2^L218M^ | ATGCAGAGCCAACCAGACAATGTGGCGTATCCCATGGAGCTACAAGCGGTTAATAAGGATGGAACAGTGGAGGTCCGTGTTCAGGGAAACGTTGACAACAGTAGCAATGAGCGGTGGGATGCAGATGTACAAAAACATGAGGTGGCGGAGGCTCAAGAGAAACCCGTGGGAGGCATCAACTTTTGGGCACCACGGGAACTGCGGCTAAATTACCGCGACTACGTGGCTGAATTTCTGGGAAACTTCGTCCTCATATATATCGCTAAGGGCGCGGTTATCACCTCACTACTTGTTCCAGATTTTGGGCTTCTCGGTCTTACGATTGGTATTGGTGTGGCTGTCACGATGGCTCTGTATGTTTCACTGGGCATCTCCGGTGGCCATCTCAACTCTGCCGTCACCGTTGGCAATGCGGTTTTCGGTGATTTCCCTTGGAGAAAAGTCCCCGGCTACATCGCGGCGCAGATGCTCGGCACTTTCCTTGGTGCTGCCTGCGCTTACGGAGTGTTTGCTGATCTCCTGAAGGCGCATGGTGGTGGTGAGTTGATTGCCTTCGGTGAAAAGGGGATTGCGGGGGTGTTTGCCATGTACCCCGCGGAAGGAAATGGTATATTTTATCCAATTTTTGCTGAACTCATTTCCACCGCAGTGATGCTGCTCTGTGTCTGCGGTATCTTTGACCCCAATAACTCTCCTGCCAAGGGGTACGAAACGGTAGCTATTGGTGCTCTTGTCTTCGTCATGGTCAACAACTTCGGCTTAGCGTCTCCCCTTGCGATGAATCCCTCACTTGATTTCGGTCCCAGGGTCTTCGGTGCGATCCTTCTCGGGGGGGAAGTTTTTTCACATGCAAATTATTATTTCTGGGTTCCACTAGTTGTTCCATTCTTTGGAGCTATCCTTGGACTTTTTCTGTACAAATATTTTCTTCCACACTAA | ATG: 652-654 |
| gene (*Trypanosoma brucei*) | TbAQP2^L84W/L118W^ | ATGCAGAGCCAACCAGACAATGTGGCGTATCCCATGGAGCTACAAGCGGTTAATAAGGATGGAACAGTGGAGGTCCGTGTTCAGGGAAACGTTGACAACAGTAGCAATGAGCGGTGGGATGCAGATGTACAAAAACATGAGGTGGCGGAGGCTCAAGAGAAACCCGTGGGAGGCATCAACTTTTGGGCACCACGGGAACTGCGGCTAAATTACCGCGACTACGTGGCTGAATTTCTGGGAAACTTCGTCTGGATATATATCGCTAAGGGCGCGGTTATCACCTCACTACTTGTTCCAGATTTTGGGCTTCTCGGTCTTACGATTGGTATTGGTGTGGCTGTCACGATGGCTTGGTATGTTTCACTGGGCATCTCCGGTGGCCATCTCAACTCTGCCGTCACCGTTGGCAATGCGGTTTTCGGTGATTTCCCTTGGAGAAAAGTCCCCGGCTACATCGCGGCGCAGATGCTCGGCACTTTCCTTGGTGCTGCCTGCGCTTACGGAGTGTTTGCTGATCTCCTGAAGGCGCATGGTGGTGGTGAGTTGATTGCCTTCGGTGAAAAGGGGATTGCGGGGGTGTTTGCCATGTACCCCGCGGAAGGAAATGGTATATTTTATCCAATTTTTGCTGAACTCATTTCCACCGCAGTGCTACTGCTCTGTGTCTGCGGTATCTTTGACCCCAATAACTCTCCTGCCAAGGGGTACGAAACGGTAGCTATTGGTGCTCTTGTCTTCGTCATGGTCAACAACTTCGGCTTAGCGTCTCCCCTTGCGATGAATCCCTCACTTGATTTCGGTCCCAGGGTCTTCGGTGCGATCCTTCTCGGGGGGGAAGTTTTTTCACATGCAAATTATTATTTCTGGGTTCCACTAGTTGTTCCATTCTTTGGAGCTATCCTTGGACTTTTTCTGTACAAATATTTTCTTCCACACTAA | TGG: 250-253  TGG: 352-354 |
| gene (*Trypanosoma brucei*) | TbAQP3W.T  (Tb927.10.14160) | ATGCAGAGCCAACCAGACAATGTGGCGTATCCCATGGAGCTACAAGCGGTTAATAAGGATGGAACAGTGGAGGTCCGTGTTCAGGGAAACGACGACAGTAGCAACCGGAAACATGAGGTGGCGGAGGCTCAAGAGGAAGTACCGGGAGGCATCAACTTTTGGGCACCACGGGAACTGCGGCTAAATTACCGCGACTACATGGGGGAGCTGCTGGGAACCTTCGTCCTGCTCTTTATGGGTAATGGTGTGGTCGCCACGGTTATCATTGATGGGAAACTGGGGTTCCTCAGCATTACGCTTGGTTGGGGCATTGCCGTCACGATGGCTCTGTATGTTTCATTGGGTATCTCGAGCGGCCACCTTAATCCCGCCGTCACCGTTGGCAATGCGGTTTTCGGTGATTTCCCTTGGAGAAAAGTCCCCGGCTACATCGCGGCGCAGATGCTCGGTGCCTTTCTTGGTGCTGCCTGCGCTTACGGAGTGTTTGCTGATCTCCTGAAGGCGCATGGTGGTGGTGAGTTGATTGCCTTCGGTGAAAAGGGGACCGCAGGTGTGTTCAGCACCTACCCAAGGGATTCAAATGGTCTATTTTCTTGTATCTTTGGTGAGTTTATATGTACGGCGATGCTATTGTTCTGTGTCTGCGGTATCTTCGACCCCAATAACTCTCCTGCCAAGGGACACGAGCCGTTGGCAGTTGGTGCTCTTGTCTTCGCCATTGGCAATAACATCGGTTACTCAACGGGTTACGCAATAAATCCGGCTCGTGACTTCGGTCCCAGGGTCTTCTCTTCTTTTCTTTATGGTGGGAAGGTGTTTTCACATGCAAATTATTATTTCTGGGTTCCACTAGTTATTCCATTGTTTGGAGGTATCTTTGGACTTTTTCTGTACAAATATTTTGTGCCACACTAA |  |
| gene (*Trypanosoma brucei*) | TbAQP3^W102I/R256L^ | ATGCAGAGCCAACCAGACAATGTGGCGTATCCCATGGAGCTACAAGCGGTTAATAAGGATGGAACAGTGGAGGTCCGTGTTCAGGGAAACGACGACAGTAGCAACCGGAAACATGAGGTGGCGGAGGCTCAAGAGGAAGTACCGGGAGGCATCAACTTTTGGGCACCACGGGAACTGCGGCTAAATTACCGCGACTACATGGGGGAGCTGCTGGGAACCTTCGTCCTGCTCTTTATGGGTAATGGTGTGGTCGCCACGGTTATCATTGATGGGAAACTGGGGTTCCTCAGCATTACGCTTGGTATGGGCATTGCCGTCACGATGGCTCTGTATGTTTCATTGGGTATCTCGAGCGGCCACCTTAATCCCGCCGTCACCGTTGGCAATGCGGTTTTCGGTGATTTCCCTTGGAGAAAAGTCCCCGGCTACATCGCGGCGCAGATGCTCGGTGCCTTTCTTGGTGCTGCCTGCGCTTACGGAGTGTTTGCTGATCTCCTGAAGGCGCATGGTGGTGGTGAGTTGATTGCCTTCGGTGAAAAGGGGACCGCAGGTGTGTTCAGCACCTACCCAAGGGATTCAAATGGTCTATTTTCTTGTATCTTTGGTGAGTTTATATGTACGGCGATGCTATTGTTCTGTGTCTGCGGTATCTTCGACCCCAATAACTCTCCTGCCAAGGGACACGAGCCGTTGGCAGTTGGTGCTCTTGTCTTCGCCATTGGCAATAACATCGGTTACTCAACGGGTTACGCAATAAATCCGGCTCTTGACTTCGGTCCCAGGGTCTTCTCTTCTTTTCTTTATGGTGGGAAGGTGTTTTCACATGCAAATTATTATTTCTGGGTTCCACTAGTTATTCCATTGTTTGGAGGTATCTTTGGACTTTTTCTGTACAAATATTTTGTGCCACACTAA | ATG: 304-306  CTT: 766-768 |
| gene (*Trypanosoma brucei*) | TbAQP3^W102I/^^R256L/Y250L^ | ATGCAGAGCCAACCAGACAATGTGGCGTATCCCATGGAGCTACAAGCGGTTAATAAGGATGGAACAGTGGAGGTCCGTGTTCAGGGAAACGACGACAGTAGCAACCGGAAACATGAGGTGGCGGAGGCTCAAGAGGAAGTACCGGGAGGCATCAACTTTTGGGCACCACGGGAACTGCGGCTAAATTACCGCGACTACATGGGGGAGCTGCTGGGAACCTTCGTCCTGCTCTTTATGGGTAATGGTGTGGTCGCCACGGTTATCATTGATGGGAAACTGGGGTTCCTCAGCATTACGCTTGGTATGGGCATTGCCGTCACGATGGCTCTGTATGTTTCATTGGGTATCTCGAGCGGCCACCTTAATCCCGCCGTCACCGTTGGCAATGCGGTTTTCGGTGATTTCCCTTGGAGAAAAGTCCCCGGCTACATCGCGGCGCAGATGCTCGGTGCCTTTCTTGGTGCTGCCTGCGCTTACGGAGTGTTTGCTGATCTCCTGAAGGCGCATGGTGGTGGTGAGTTGATTGCCTTCGGTGAAAAGGGGACCGCAGGTGTGTTCAGCACCTACCCAAGGGATTCAAATGGTCTATTTTCTTGTATCTTTGGTGAGTTTATATGTACGGCGATGCTATTGTTCTGTGTCTGCGGTATCTTCGACCCCAATAACTCTCCTGCCAAGGGACACGAGCCGTTGGCAGTTGGTGCTCTTGTCTTCGCCATTGGCAATAACATCGGTTACTCAACGGGTCTCGCAATAAATCCGGCTCTTGACTTCGGTCCCAGGGTCTTCTCTTCTTTTCTTTATGGTGGGAAGGTGTTTTCACATGCAAATTATTATTTCTGGGTTCCACTAGTTATTCCATTGTTTGGAGGTATCTTTGGACTTTTTCTGTACAAATATTTTGTGCCACACTAA | ATG: 304-306  CTC: 748-750  CTT: 766-768 |
