# Supplementary material for: Positively selected modifications in the pore of TbAQP2 allow pentamidine to enter Trypanosoma brucei
Source: eLife. 2020 Aug 11;9:e56416. doi: 10.7554/eLife.56416 (PMC7473772; doi:10.7554/eLife.56416)
Supplement: Supplementary file 3. [file elife-56416-supp3.docx]

Supplemental File 3. Synthesis of new inhibitors.

**Chemistry of new compounds from Paul O’Neill laboratory.**

**1,2-Bis (4-cyanophenoxy) ethane** (Ethamidine Precursor) **(1a)** ^1^

Sodium (0.16 g, 6.96 mmol) was added portionwise to anhydrous EtOH (4.0 mL) under an atmosphere of nitrogen. After dissolution of the sodium pieces, a solution of 4-cyanophenol (0.75 g, 6.38 mmol) dissolved in anhydrous EtOH (4.0 mL) was added followed by dropwise addition of 1,2-dibromoethane (0.28 mL, 3.19 mmol). The reaction mixture was allowed to stir at reﬂux under a nitrogen atmosphere for 3 days after which the mixture was cooled, ﬁltered, the solid washed with water and dried under vacuum. Puriﬁcation by column chromatography eluting with DCM: hexane (8:2) gave the desired dinitrile ethamidine precursor (**1a**) as a white solid (1.42 g, 84%). Mp 211-212°C; ^1^H NMR (CDCl_3_, 400MHz) δ 7.61 (d, 4H, *J* = 9.0 Hz, ArH), 7.01 (d, 4H, *J* = 9.0 Hz, ArH), 4.39 (s, 4H, CH_2_); ^13^C NMR (CDCl_3_, 100MHz) δ 161.6, 134.1, 118.9, 115.3, 104.7, 66.4; ν_max_ (NujOI) /cm^-1^ 3326 (C-O-C), 3033 (ArH), 2898 (OH), 2223 (CN), 1602 (Ar), 1509 (Ar), 1247 (C-O-C); *m/z* (CI) 282 ([M+NH_4_]^+^), found 282.12433, C_16_H_16_O_2_N_3_ requires 282.12424; anal. Found C 72.37, H 4.51, N 10.54, C_16_H_12_O_2_N_2_ requires C 72.71, H 4.57, N 10.60.

**4,4'-(ethane-1,2-diylbis(oxy))dibenzimidamide dihydrochloride dihydrate** (Ethamidine, Compound CHI/1/30/1) (**2**) ^1^

(0.51 g, 1.92 mmol) of **1a** was dissolved in a mixture of anhydrous benzene (54 mL) and EtOH (2.90 mL), cooled to 0 °C and saturated with HCl gas. The mixture was sealed and allowed to stir at room temperature for 3 days after which anhydrous Et_2_O (28 mL) was introduced and the mixture was allowed to stir for 10 minutes. The solids were ﬁltered under nitrogen and dissolved in a mixture of anhydrous EtOH (36 mL) and EtOH.NH_3_ (36 mL). The mixture was heated overnight (50 ^o^C), cooled to room temperature and reduced by half *in vacuo*. Ether was added to precipitate the solid which was ﬁltered, washed and dried under vacuum. Puriﬁcation by recrystallisation (2N HCl) gave the desired compound **2** as ﬁne white needles (0.54 g, 70%). Mp 333°C; ^1^H NMR (MeOD, 400MHz) δ 7.85 (d, 4H, *J* = 9.0 Hz, ArH), 7.23 (d, 4H, *J* = 9.0 Hz, ArH), 4.52 (s, 4H, CH_2_); ^13^C NMR (MeOD, 100MHz) δ 167.9, 165.3, 131.5, 121.7, 116.7, 68.5; ν_max_ (Nujol) /cm^-1^ 3362 (NH), 3037 (ArH), 2940 (C-H), 1658 (C=N-H), 1606 (Ar), 1505 (Ar), 1245 (C-0-C); *m/z* (ESP) 299 ([M-H]^-^); anal. Found C 47.54 H 5.62 N 14.30, C_16_H_24_N_4_O_4_Cl_2_ requires C 47.18, H 5.94, N 13.76.

**4, 4’-(propane-1,3-diylbis(oxy))dibenzonitrile** (Propamidine Precursor) (**1b**)^1^

Sodium (0.16 g, 6.96 mmol) was added portionwise to anhydrous EtOH (4.0 mL) with stirring under an atmosphere of nitrogen. After dissolution of Na, a solution of 4-cyanophenol (0.75 g, 6.38 mmol) dissolved in dry ethanol (4.0 mL) was added followed by dropwise addition of 1,3-dibromopropane (0.32 mL, 3.19 mmol). The reaction mixture was allowed to stir at reﬂux under a nitrogen atmosphere for 3 days after which the mixture was cooled, ﬁltered, the solid washed with water and dried under vacuum. Puriﬁcation by column chromatography eluting with DCM: hexane (8:2) gave the desired compound **1b** as a white solid (1.40 g, 79%). Mp 190-191°C; ^1^H NMR (CDCl_3_, 400MHz) δ 7.59 (d, 4H, *J* = 8.5 Hz, ArH), 6.96 (d, 4H, *J* = 8.5 Hz, ArH), 4.20 (t, 4H, *J* = 6.0 Hz, CH_2_), 2.32 (m, 2H, CH_2_); ^13^C NMR (CDCl_3_, 100MHz) δ 161.9, 134.0, 119.1, 115.1, 104.2, 64.4, 28.8; ν_max_ (Nujol) /cm^-1^ 3104 (Ar-H), 2823 (C-H), 2221 (C≡N), 1604 (Ar), 1509 (Ar), 1253 (C-O); *m/z* (CI) 296 ([M+NH_4_]^+^), found 296.14037, C_17_H_18_N_3_O_2_ requires 296.13992; anal. Found C 73.22, H 5.13, N 10.03, C_17_H_14_N_2_O_2_ requires C 73.37, H 5.07, N 10.07.

**4,4’-(propane-1,3-diyl*bis*(oxy))dibenzimidamide dihydrochloride dihydrate** (Propamidine, Compound CHI/1/25/5) (**3**)^1^

(0.50 g, 1.79 mmol) of **1b** was dissolved in a mixture of anhydrous benzene (55 mL) and anhydrous ethanol (3.0 mL), cooled to 0 ^o^C and saturated with HCl gas. The mixture was sealed and allowed to stir at room temperature for 3 days after which ether (30 mL) was added and the mixture was allowed to stir for 10 minutes. The solids were ﬁltered under nitrogen and dissolved in a mixture of anhydrous EtOH (36 ml) and EtOH.NH_3_ (36 mL). The mixture was heated overnight (50 °C), cooled to room temperature and reduced by half *in vacuo*. Et_2_O (15 mL) was added to precipitate the solid which was ﬁltered, washed and dried under vacuum. Puriﬁcation by recrystallisation (2N HCl) gave the desired compound **3** as ﬁne white needles (0.59 g, 78%). Mp 200°C; ^1^H NMR (MeOD, 400MHz) δ 7.82 (d, 4H, *J* = 9.0 Hz, ArH), 7.19 (d, 4H, *J* = 9.0 Hz, ArH), 4.34 (t, 4H, *J* = 6.0 Hz, CH_2_), 2.46 (m, 2H, CH_2_); ^13^C NMR (MeOD, 100MHz) δ 167.9, 165.5, 131.5, 121.5, 121.4, 116.6, 66.4, 30.3; ν_max_ (Nujol) /cm^-1^ 3280 (N-H), 3038 (Ar-H), 2929 (C-H), 1504 (Ar), 1606 (Ar), 1240 (C-O-C); *m/z* (ESP) 313 ([M-H]^-^); anal. Found C 48.60, H 6.10, N 13.25, C_17_H_26_N_4_O_4_Cl_2_ requires C 48.46, H 6.22, N 13.30.

**4,4’-(Butane-1,4-diylbis(oxy))dibenzonitrile** (Butamidine precursor) (**1c**)^1^

Sodium (0.10 g, 4.35 mmol) was added portionwise to dry EtOH (4.0 mL) stirring under an atmosphere of nitrogen. After dissolution of sodium, a solution of 4-cyanophenol (0.47 g, 3.95 mmol) dissolved in dry ethanol (4.0 mL) was added followed by dropwise addition of 1,4-dibromobutane (0.24 mL, 1.98 mmol). The reaction mixture was allowed to stir at reﬂux under a nitrogen atmosphere for 3 days after which the mixture was cooled, ﬁltered, the solid washed with water and dried under vacuum. Purification by column chromatography eluting with DCM: hexane (8:2) gave the desired compound **1c** as a white solid (1.03 g, 89%). Mp 174°C; 'H NMR (CDCI_3_, 400MHz) δ 7.59 (d, 4H, *J* = 8.9 Hz, ArH), 6.93 (d, 4H, *J* = 8.9 Hz, ArH), 4.08 (m, 4H, CH_2_), 2.01 (m, 4H, CH_2_); ^13^C NMR (CDCl_3_, 100MHz) δ 162,1, 134.0, 119.1, 115.1, 104.0, 67.7, 25.7; ν_max_ (Nujol) /cm^-1^ 3332 (C-O-C), 3033 (Ar-H). 2956 (C-H), 2219 (C≡N), 1604 (Ar). 1506 (Ar), 1251 (C-O-C); *m/z* (CI) 310 ([M+NH_4_]^+^) found 310.15532, C_18_H_20_N_3_O_2_ requires 310.15555; anal. Found C 74.03, H 5.55, N 9.55, C_18_H_16_N_2_O_2_ requires 73.95, H 5.52, N 9.58.

**4,4’-(Butane-l ,4-diylbis(oxy))dibenzimiamide dihydrochloride dihydrate** (Butamidine, Compound CHI/1/41/1) (**4**)^1^

Compound **1c** (0.42 g, 1.44 mmol) was dissolved in a mixture of anhydrous benzene (46 mL) and anhydrous ethanol (2.50 mL), cooled to 0 °C and saturated with HCl gas. The mixture was sealed and allowed to stir at room temperature for 3 days after which anhydrous Et_2_O (40 mL) was introduced and the mixture was allowed to stir for 10 minutes. The solids were ﬁltered under nitrogen and dissolved in a mixture of anhydrous EtOH (34 mL) and EtOH.NH_3_ (34 mL). The mixture was heated overnight (50 °C), cooled to room temperature and reduced by half *in vacuo*. Ether (15 mL) was added to precipitate the solid which was ﬁltered, washed and dried under vacuum. Puriﬁcation by recrystallisation (2N HCl) gave the desired compound **4** as ﬁne white needles (0.46 g, 73%). Mp 286-287°C', ‘H NMR (MeOD, 400MHz) δ, 7.80 (d, 4H, *J* = 9.0 Hz, ArH), 7.14 (d, 4H, *J* = 9.0Hz, ArH), 4.19 (m, 4H, CH_2_), 2.02 (m, 4H, CH_2_); ^13^ CNMR (MeOD, 100MHz) δ 165.7, 131.4, 121.2, 116.7, 69.7, 27.2; ν_max_ (Nujol) /cm^-1^ 3370 (N-H), 3129 (Ar-H), 2884 (C-H), 1650 (C≡N), 1606 (Ar), 1508 (Ar), 1257 (C-O); *m/z* (ESP) 327 ([M+H]^+^); anal. Found C 49.87, H 6.46, N 12.67, C_18_H_28_N_4_O_4_Cl_2_ requires C 49.66, H 6.48, N 12.87.

**4-(4-phenoxybutoxy) benzonitrile**

Sodium (0.16g, 6.96 mmol) was added dropwise to dry ethanol (5 mL) and dissolved under a nitrogen atmosphere. To this a solution of 4-cyanophenol (0.53g, 4.47 mmol) dissolved in anhydrous ethanol (5ml) was added followed by addition of 1,4-dibromobutane (0.53 mL, 4.47 mmol). The reaction mixture was allowed to stir at reflux and monitored by TLC. After consumption of 4-cyanophenol, the reaction mixture was allowed to cool to room temperature. In a separate ﬂask sodium (0 16 g, 6.96 mmol) was added portionwise to ethanol (5 mL) stirring under nitrogen. A solution of phenol (0.42 g, 4.47 mmol) in ethanol (5ml) was added and stirred for 10 minutes. This mixture was added dropwise to the cooled mixture and allowed to stir under reﬂux for 3 days after which the mixture was cooled, ﬁltered, the solid washed with water and dried under vacuum. Puriﬁcation by column chromatography eluting with DCM: hexane (8:2) gave the desired compound 4-(4-phenoxybutoxy) benzonitrile as a white solid (0.98 g, 82%). Mp 130°C; ^1^H NMR (CDCl_3,_ 400MHZ) δ 7.57 (d, 2H, *J* = 8.9 Hz, ArH), 7.28 (d, 1H, *J* = 7.5 Hz, ArH), 7.26 (d, 1H, *J* = 8.1 Hz, ArH), 6.92 (m, 5H, ArH), 4.08 (t, 2H, *J* = 5.9 Hz, CH_2_), 4.03 (t, 2H, *J* = 5.9 Hz, CH_2_), 1.99 (m, 4H, CH_2_); ^13^C NMR (CDCl_3_, 100MHz) δ 162.6, 159.2, 134.3, 129.8, 121.1, 115.5, 114.8, 104.3, 68.3, 67.5, 26.2; ν_max_ (Nujol) /cm^-1^ 3043 (Ar-H), 2884 (C-H), 2219 (C≡N), 1602 (Ar), 1504 (Ar), 1247 (C-O); *m/z* (CI) 285 ([M+NH_4_]^+^), found 285.16020, C_17_H_21_N_2_O_2_ requires 285.16031; anal. Found C 76.40, H 6.46, N 5.44, C_17_H_17_NO_2_ requires C 76.38, H 6.40, N 5.24.

**4-(4-Phenoxybutoxy)benzimidamide hydrochloride hydrate** (Compound CHI/1/69/1)

4-(4-phenoxybutoxy) benzonitrile (0.27 g, 1.01 mmol) was dissolved in a mixture of anhydrous benzene (100 mL) and ethanol (1.60 mL), cooled to 0 °C and saturated with HCl gas. The mixture was sealed and allowed to stir at room temperature for 3 days after which anhydrous Et_2_O (16 mL) was introduced and the mixture was allowed to stir for an additional 10 minutes. The solids were ﬁltered under nitrogen and dissolved in a mixture of anhydrous EtOH (20 mL) and anhydrous EtOH.NH_3_ (20 mL). The mixture was heated overnight (50 ^o^C), cooled to room temperature and reduced by half *in vacuo*. Ether (30 mL) was added to precipitate the solid which was ﬁltered, washed and dried under vacuum. Puriﬁcation by recrystallisation (2N HCl) gave the desired compound 4-(4**-**Phenoxybutoxy)benzimidamide hydrochloride hydrate as fine white needles (0.28 g, 82%). Mp 134-135°C; ^1^H NMR (DMSO, 400MHz) δ 9.28 (s, 2H, NH;), 9.08 (s, 2H, NHZ), 7.86 (d, 2H, *J* = 9.0 Hz, ArH), 7.29 (d, 1H, *J* = 7.0 HZ, ArH), 7.27 (d, 1H, *J* = 7.2 Hz, ArH), 7.16 (d, 2H, *J* = 9.0 Hz, ArH), 6.93 (d, 3H, *J* = 7.8 Hz, ArH), 4.16 (t, 2H, *J* = 5.9 Hz, CH_2_), 4.03 (t, 2H, *J* = 5.9 Hz, CH_2_), 1.89 (m, 4H, CH_2_); ^13^C NMR (DMSO, 100MHZ) δ 165.0, 163.3, 158.9, 130.5, 129.8, 120.7, 119.6, 115.1, 114.7, 68.1, 67.2, 25.6, 25.5; ν_max_ (Nujol) /cm^-1^ 3288 (N-H), 1656 (C=N-H), 1604 (Ar), 1506 (Ar), 1234 (C-O-C); *m/z* (ESP) 285 ([M+H]^+^), found 285.1603, C_17_H_24_N_2_O_2_ requires 285.1599; anal. Found C 59.50, H 6.75, N 8.33, C_17_H_23_N_2_O_3_Cl requires C 60.26, H 6.84, N 8.27.

**4-(5-(*p*-Tolyloxy)pentyloxy)benzonitrile**

Sodium (0.12 g, 5.22 mmol) was added portionwise to anhydrous ethanol (4.0 mL) and dissolved under a nitrogen atmosphere. To this a solution of 4-cyanophenol (0.57 g, 4.79 mmol) dissolved in anhydrous ethanol (4.0 mL) was added followed by dropwise addition of 1,5-dibromopentane (0.65 mL, 4.79 mmol). The reaction mixture was allowed to stir at reﬂux and monitored by TLC. After consumption of 4-cyanophenol the reaction mixture was cooled to room temperature. In a separate flask, sodium (0.57 g, 4,79 mmol) was added portionwise to anhydrous EtOH (4.0 ml) with stirring under nitrogen. To this, a solution of *p*-cresol (0.5 ml, 4.79 mmol) in anhydrous EtOH (4.0 ml) was added and stirred for 10 minutes. This mixture was added dropwise to the cooled mixture and stirred under reflux for 3 days after which the mixture was cooled, filtered and the solid washed with water and dried under vacuum. Purification by column chromatography eluting with DCM: hexane (8:2) gave the desired compound as a white solid(1.02 g, 72%). Mp 133°C; ^1^H NMR (CDCl_3_, 400MHz) δ 7.57 (d, 2H, *J* = 9 Hz, ArH), 7.07 (d, 2H, *J* = 8.6 Hz, ArH), 6.93 (d, 2H, *J =* 9.0 Hz, ArH), 6.79 (d, 2H, *J* = 8.6 Hz, ArH) 4.02 (t, 2H, *J* = 6.4 Hz, CH_2_) 3.96 (t, 2H, *J* = 6.4 Hz, CH_2_), 2.28 (s, 3H, CH3), 1.86 (m, 4H, CH_2_), 1.64 (m, 2H, CH_2_); ^13^C NMR (CDCl_3_, 100MHz) δ 162.7, 157.2, 134.3, 130.3, 130.2, 115.5, 114.7, 104.1, 68.5, 68.0, 29.4, 29.1, 23.0, 20.8; ν_max_ (Nujol) /cm^-1^ 3322 (C-O-C), 3031 (Ar-H), 2921 (C-H), 2223 (C≡N), 1602 (Ar), 1506 (Ar), 1234 (C-O-C); *m/z* (CI) 313 ([M+NH_4_]^+^), found 313.19092, C_19_H_25_N_2_O_2_ requires 313.19162; anal. Found C 77.19, H 7.13, N 5.02, C_19_H_21_NO_2_ requires C 77.26, H 7.17, N 4.74.

**4-(5-(*p*-tolyloxy)pentyyloxy)benzimidamide hydrochloride hydrate** (Compound CHI/1/72/1)

4-(5-(*p*-Tolyloxy)pentyloxy)benzonitrile (0.27 g, 0.91 mmol) was dissolved in a mixture of anhydrous benzene (100 mL) and anhydrous ethanol (1.60 mL), cooled to 0 ^o^C and saturated with HCl gas. The mixture was sealed and allowed to stir at room temperature for 3 days after which anhydrous Et_2_O (20 mL) was introduced and the mixture was allowed to stir for 10 minutes. The solids were ﬁltered under nitrogen and dissolved in a mixture of anhydrous EtOH (20 mL) and EtOH.NH_3_ (20 mL). The mixture was heated overnight (50 °C), cooled to room temperature and reduced by half *in vacuo*. Ether (30 mL) was added to precipitate the solid which was ﬁltered, washed and dried under vacuum. Puriﬁcation by recrystallisation (2N HCl) gave the desired compound as ﬁne white needles (0.25 g, 75%). Mp 132°C; ^1^H NMR (DMSO, 400MHZ) δ 7.84 (d, 2H, *J* = 9.1 Hz, ArH), 7.15 (d, 2H, *J* = 9.1 Hz, ArH), 7.06 (d, 2H, *J* = 8.4 Hz, ArH), 6.80 (d, 2H, *J* = 8.4 Hz, ArH), 4.11 (t, 2H, *J* = 6.4 Hz, CH_2_), 3.93 (t, 2H, *J* = 6.4 Hz, CH_2_), 2.22 (s, 3H, CH_3_), 1.78 (m, 4H, CH_2_), 1.56 (m, 2H, CH_2_); ^13^C NMR (DMSO, 100MHz) δ 165.0, 163.3, 158.9, 130.5, 130.1, 115.1, 114.5, 79.5, 79.3, 79.0, 20.4; ν_max_ (Nujol) /cm^-1^ 3430 (NH), 3309 (C-O-C), 3093 (Ar-H), 1658 (C=N-H), 1606 (Ar), 1508 (Ar), 1245 (C-O-C); *m/z* (ESP) 313 ([M+H]^+^), found 313.1916, C_19_H_25_N_2_O_2_ requires 313.1907; anal. Found C 62.19, H 7.42, N 7.66, C_19_H_27_ClN_2_O_3_ requires C 62.20, H 7.42, N 7.66.

**Synthesis of ER 1004**

**4-bromobenzothioamide**

Triethylamine (3.8 mL) was added to a solution of 4-bromobenzonitrile (5 g, 27.5 mmol) in pyridine (17 mL). The solution was cooled to 10 ^o^C and H_2_S (g) was bubbled through for 15 min. The resulting green solution was allowed to stir overnight (17 h). Nitrogen was bubbled through for 1 h to remove any excess H_2_S. Water (27 mL) was added and the mixture was stirred for 10 min, a further portion of water (62 mL) was added and the pale yellow suspension left stirring overnight. The precipitate was filtered and rinsed with water to afford the title compound as bright yellow crystals (5.52 g, 93%). ^1^H NMR (d_6_-acetone, 400MHz) 9.07 (bs, 1H, NH), 8.92 (s, 1H, NH), 7.94 (dd, 2H, *J* = 2.0, 6.5 Hz, ArH), 7.62 (dd, 2H, *J* = 2.0, 6.5 Hz, ArH); ^13^C NMR (d_6_-acetone, 100MHz) 201.9, 140.2, 132.3, 130.4, 126.5; *m/z* (CI) 216 (100%, [M^+^])

**2,4-bis(4-bromophenyl)thiazole**

2,4'-dibromoacetophenone (1 g, 3.60 mmol) was added to a solution of 4-bromobenzothioamide (777 mg, 3.60 mmol) in EtOH (15 mL) and warmed to 45 ^o^C for 1 h. The mixture was cooled to room temperature and left for 30 min before filtering. The precipitate was washed with EtOH: water (3:1, 10 mL) and dried to afford the thiazole as a pale solid (1.33 g, 94%). ^1^H NMR (CDCl_3_, 250MHz) 7.89 (d, 2H, *J* = 8.5 Hz, ArH), 7.85 (d, 2H, *J* = 8.5 Hz, ArH), 7.59 (d, 2H, *J* = 5.5 Hz, ArH), 7.56 (d, 2H, *J* = 5.5 Hz, ArH), 7.47 (s, 1H, CH); *m/z* (CI) 396 (10%, [M+H]^+^).

**4,4'-(thiazole-2,4-diyl)dibenzonitrile**

A suspension of 2,4-bis(4-bromophenyl)thiazole (1 g, 2.53 mmol) and CuCN (906 mg, 10.12 mmol) in anhydrous DMF (15 mL) were heated to reflux for 21 h. On cooling, the reaction mixture was poured into aqueous NH_4_OH (10%, 50 mL) and extracted with CHCl_3_ (100 mL). Both layers were filtered to remove the dark precipitate. The organic layer was washed with water (2 x 50 mL), brine (50 mL) and dried MgSO_4_. Removal of solvent gave a dark oily solid. Purification by column chromatography eluting with CHCl_3,_ afforded the title compound as a pale solid (361 mg, 50%). ^1^H NMR (CDCl_3_, 400MHz) 8.15 (d, 2H, *J* = 8.5 Hz, ArH), 8.11 (m, 2H, ArH), 7.77 (d, 2H, *J* = 8.5 Hz, ArH), 7.73 (t, 2H, *J* = 3.0 Hz, ArH), 7.26 (s, 1H, CH); ^13^C NMR (CDCl_3_, 100MHz) 166.5, 137.4, 133.2, 130.1, 127.3, 119.1, 117.1, 114.2, 112.4; *m/z* (CI) 288 (100 %, [M+H]^+^).

**4,4'-(thiazole-2,4-diyl)dibenzimidamide (ER1004)**

#### The Garigipati Reaction is a little known reaction which effects the conversion of hindered nitriles to unsubstituted amidines in a mild and effective manner ^2, 3^. This is an efficient one step transformation involving direct nucleophilic addition of an amine to a nitrile, affording the corresponding amidine (Scheme 1).

**Scheme 1.**

The alkylchloroaluminium amides are effectively generated from trimethyl aluminium and ammonium chloride and the intermediate aluminium complex is easily hydrolyzed by water adsorbed on silica gel (Scheme 2).

###

**Scheme 2.**

The freshly prepared alkylchloroaluminum reagents (4 mL, 0.67 M, 2.7 mmol) were added to 4,4'-(thiazole-2,4-diyl)dibenzonitrile (78 mg, 0.27 mmol) in anhydrous toluene (1 mL) and heated to 80 ^o^C overnight under nitrogen. On cooling, the aluminium complex was decomposed by pouring into a slurry of silica gel (2 g) in CHCl_3_. The mixture was stirred for 5 min before filtering, the filter cake was washed with MeOH (20 mL). Removal of solvent gave the crude amidine as a pale solid in quantitative yield. The crude product (100 mg) was purified by reverse phase HPLC using a YMC-pack ODS-A column (250 x 20 mm I.D, 5 µM) eluting with CH_3_CN: Water 0.1% TFA (20-80 % gradient over 20 min). Removal of solvent afforded the desired compound as an off-white solid (38 mg, 22%). Mp 271-272 ^o^C; ^1^H NMR (DMSO, 400MHz) 9.32 (bs, 6H, NH & NH_2_), 8.54 (s, 1H, CH), 8.25 (d, 2H, *J* = 8.5 Hz, ArH), 8.22 (d, 2H, *J* = 8.5 Hz, ArH), 7.92 (d, 2H, *J* = 8.5 Hz, ArH), 7.90 (d, 2H, *J* = 8.5 Hz, ArH); ^13^C NMR (DMSO, 100MHz) 165.5, 165.4, 154.4, 138.7, 137.4, 129.6, 129.2, 127.9, 126.9, 126.8, 119.6; *m/z* (ES) 322 (88 % [M+H]^+^); Found (ES) 322.1121 C_17_H_16_N_5_S requires 322.1126; anal. Found C 46.05, H 3.10, N 12.44, S 5.77, C_21_H_18_N_5_O_4_F_6_S requires C 45.91, H 3.12, N 12.74, S 5.83.

References

1. R. R. Tidwell, S. K. Jones, J. D. Geratz, K. A. Ohemeng, M. Cory and J. E. Hall, *Journal of Medicinal Chemistry*, 1990, **33**, 1252-1257.

2. Moss, R.A., et al., *Conversion of Obstinate Nitriles to Amidines by Garigipatis Reaction.* *Tetrahedron Letters*, 1995. **36**(48): p. 8761-8764.

3. Garigipati, R.S., *An Efficient Conversion of Nitriles to Amidines.* Tetrahedron Letters, 1990. **31**(14): p. 1969-1972.

Chemistry of new compounds from David Boykin laboratory.

**General procedure for conversion of nitriles into amidine hydrochlorides (Method A).**

To a cold and stirred suspension of the nitrile or dinitrile (0.001 mol) in 15 ml dry THF was added 6.0 ml, (0.006 mol) LiN(TMS)_2_ (1 M in THF), stirred for 24 h, cooled, acidified carefully with saturated ethanolic-HCl, the precipitated white solid stirred for 2 h, solvent removed under reduced pressure, diluted with ether, filtered. The collected solid was added to 10 ml ice water, basified with 2M NaOH, the precipitate was filtered, washed with water and air dried. The solid was suspended in anhydrous ethanol (15 ml) and 5 ml saturated ethanolic-HCl and stirred for 6 h, ethanol was distilled off, triturated with dry ether and filtered. The solid was dried under reduced pressure at 80^0^C for 12 h to yield (70-75%) amidine hydrochloride.

**General procedure for conversion of nitriles into amidine hydrochlorides (Method B).**

A suspension of the nitrile or dinitile (0.001 mole) in 20 ml saturated ethanol-HCl was stirred for 4 days in a closed stoppered flask, followed by precipitation with anhydrous ether. The precipitated light yellow solid imidate ester dihydrochloride was filtered and dried under reduced pressure for 3 h to yield (65% -70%) amidine hydrochloride. The imidate ester dihydrochloride (0.0005 mole) in 20 ml anhydrous ethanol was saturated with ammonia(g) or 0.03 equivalents of ethylene diamine stirred in ethanol (at reflux) for 12 h, solvent removed, 20 ml ice water added, basified to pH 10 with aqueous 2N NaOH, filtered, and washed with water. The precipitated solid was dried in air, suspended in 10 ml of saturated ethanolic-HCl, and stirred for 2 h. The solvent removed, dry ether 20 ml added, filtered, washed with ether and dried under reduced pressure for 12 h. The product amidine hydrochloride was obtained as yellow solid 60-66% yield.

**4-(5-(4-methoxyphenyl) furan-2-yl) benzimidamide hydrochloride (DB 607)**

A mixture of 5-(4-cyanophenyl)-2-bromo furan^1^ (1.23 g, 0.005 mole) and 4-methoxyphenyl boronic acid (0.93 g, 0.006 mole) in 75 ml dioxane under nitrogen was added K_2_CO_3_ (1.38 g, 0.01 mole, in 5 ml H_2_O), followed by Pd(PPh_3_)_4_ 0.12 g (0.0001 mole) and the solution was heated under reflux for 12-24 h (tlc monitored). The solvent was removed under reduced pressure, solid filtered, washed with hexane and dried in air. The solid was suspended in DCM (100 ml), filtered through celite, concentrated under reduced pressure, triturated with ether: hexane (2:1), and filtered to yield 4-(5-(4-methoxyphenyl)furan-2-yl)benzonitrile as a yellow brown solid 0.76 g (74%) mp 250-2 °C dec ; ^1^H NMR (DMSO-d_6_): 7.94 (d, 2H, J= 10.4 Hz), 7.85 (d, 2H, J= 10.4 Hz), 7.78 (d, 2H, J= 10.8 Hz), 7.28 (d, 1H, J= 4.4 Hz), 7.02 (d, 2H, J= 10.8 Hz), 6.97 (d, 1H, J= 4.4 Hz), 3.81 (s, 3H); ^13^C NMR (DMSO-d_6_): 159.3, 154.5, 150.0, 134.1, 132.9, 125.4, 123.5, 122.5, 119.0, 114.4, 111.8, 108.8, 106.9, 55.2; MS: HRMS-ESI-POS: Calcd. for C_18_H_14_NO_2_ *m/z* 276.1024 (M^+^+1), found *m/z* 276.1021.

The amidine hydrochloride was obtained as yellow solid (Method A) 0.24 g (74%) ; mp >318°C dec ; ^1^H NMR (DMSO-d_6_): 9.41 (brs, 2H), 9.1 (brs, 2H), 8.01 (d, 2H, J= 8.4 Hz), 7.92 (d, 2H, J= 8.4 Hz), 7.82 (d, 2H, J= 8.4 Hz), 7.324 (d, 1H, J= 3.6 Hz), 7.04 (d, 2H), 7.02 (d, 1H, J= 3.6 Hz), 3.82(s, #H); ^13^C NMR (DMSO-d_6_): 164.9, 159.2, 154.3, 150.2, 134.8, 128.8, 125.5, 125.4, 123.0, 122.5, 114.4, 111.4, 106.8, 55.2; ; MS: HRMS-ESI-POS.: Calcd. for C_18_H_17_N_2_O_2_ *m/z* 293.1289 (M^+^+1), found *m/z* 293.1274; Anal. calcd. for C_18_H_16_N_2_O_2_-HCl: C, 65.75; H, 5.21; N, 8.52; Found: C, 65.78; H, 5.23; N, 8.44.

**4,4'-(thiophene-2,4-diyl) dibenzimidamide dihydrochloride (DB 1077)**

A mixture of 2, 4-dibromothiophene 1.21 g (0.005 mole), 4-cyanophenylboronic acid 1.75 g (0.012 mole) following procedure for DB 607, yielded 2,4-(4-cyanophenyl)thiophene as a yellow solid, 0.86 g (72%) mp >220°C dec ; ^1^H NMR (CDCl_3_): 7.75-7.21 (m, 8H), 7.71 (d, 1H, J= 1.2 Hz), 7.64 (d, 1H, J= 1.2 Hz); ^13^C NMR (CDCl_3_): 143.6, 141.6, 139.4, 138.0, 132.9, 132.8, 126.8, 126.1, 123.8, 123.7, 118.8, 118.6, 111.3, 111.1; MS: HRMS-ESI-POS: Calcd. for C_14_H_11_N_2_S *m/z* 239.0642 (M^+^+1), found *m/z* 239.0639.

The diamidine hydrochloride was obtained as yellow solid (Method A) 0.32 g (74%) mp>300°C dec ; ^1^H NMR (DMSO-d_6_): 9.55 (brs, 2H), 9.54 (brs, 2H), 9.31 (brs, 4H), 8.42 (s, 1H), 8.32 (s, 1H), 8.09 (d, 2H, J= 8.4 Hz), 8.03-7.97 (m, 6H); ^13^C NMR (DMSO-d_6_): 164.9, 164.8, 142.5, 140.9, 139.5, 138.3, 129.1, 128.8, 126.6, 126.3, 126.2, 125.4, 125.0, 124.8; MS: HRMS-ESI-POS.: Calcd. for C_18_H_18_N_4_S *m/z* 161.0626 (M^+^+2)/2, found *m/z* 161.0621; Anal. calcd. for C_18_H_16_N_2_S-2HCl-2H_2_O: C, 50.35; H, 5.16; N, 13.05; Found: C, 50.52; H, 5.23; N, 13.22.

**3,3'-(furan-2,5-diyl bis (4,1-phenylene)) dipropanimidamide dihydrochloride (DB 1061)**

To a mixture of 4-bromophenyl propionitrile 0.63 g (0.003 mole) and 2, 5-bis (tributylstannyl) furan in 30 ml anhydrous dioxane under nitrogen was added Pd(PPh_3_)_4_ 0.14 g (0.00012 mole) and the solution was heated under reflux for 12 h (tlc monitored). The solvent was removed under reduced pressure, the solid was filtered, washed with hexane and dried in air. The solid was suspended in DCM (50 ml), stirred 2 h with 20 ml 10% KF (aqueous), the organic layer separated, filtered through celite dried over anhydrous MgSO_4_, filtered, concentrated, triturated with hexane and the solid filtered was filtered to yield 0.34 g (70%) of 3,3'-(furan-2,5-diylbis(4,1-phenylene))dipropanenitrile as a yellow solid mp 120-2°C dec; ^1^H NMR (CDCl_3_): 7.73 (d, 4H, J= 8.4 Hz), 7.30 (d, 4H, J= 8.4 Hz),7.28 (s, 2H), 3.0 (t, 4H, J= 7.6 Hz), 2.66 (t, 4H, J= 7.6 Hz); ^13^C NMR (CDCl_3_): 153.2, 137.2, 130.0, 128.9, 124.4, 119.2, 107.2, 31.5, 19.5; MS: HRMS-ESI-POS: Calcd. for C_22_H_18_N_2_ONa *m/z* 349.1317 (M^+^+Na), found *m/z*  349.1332**.**

The diamidine dihydrochloride was obtained using Method B: 0.14 g (60%) mp>300°C dec ; ^1^H NMR (DMSO-d_6_): 9.13 (brs, 4H), 8.74 (brs, 4H), 7.64 (d, 4H, J= 8.4 Hz), 7.49 (s, 2H), 7.33 (d, 4H, J=8.4 Hz), 3.0 (t, 4H, J= 7.2 Hz), 2.74 (d, 4H, J= 7.2 Hz); ^13^C NMR (DMSO-d_6_): 170.6, 142.7, 139.4, 132.4, 129.6, 125.8, 125.2, 33.7, 32.03; MS: HRMS-ESI-POS: Calcd. for C_22_H_26_N_4_O *m/z* 181.1053 (M^+^+2)/2, found *m/z* 181.1048; Anal. calc. for C_22_H_24_N_4_O-2HCl-2H_2_O: C, 56.29; H, 6.44; N, 11.93; Found: C, 56.35; H, 6.54; N, 11.86.

**2,5-bis(4-(2-(4,5-dihydro-1H-imidazol-2-yl) ethyl) phenyl) furan dihydrochloride (DB 1062)**

Similarly, 0.245 g (0.005 mole) of the above imidate ester in 20 ml of anhydrous ethanol was allowed to react under reflux (12 h) with 0.06 g (0.0015 mole) ethylene diamine. The solvent was removed under reduced pressure, diluted with water, solid was filtered, dried and converted to dihydrochloride using ethanolic-HCl to yield a yellow solid, 0.15 (62%), mp >325 ^o^C, H NMR (DMSO-d_6_): 8.29 (br, 4H), 8.74 (brs, 4H), 7.77 (d, 4H, J= 8.4 Hz), 7.34 (s, 2H), 7.05 (d, 4H, J=8.4 Hz), 3.0 (t, 4H, J= 7.2 Hz), 2.74 (d, 4H, J= 7.2 Hz); ^13^C NMR (DMSO-d_6_): 170.2, 152.4, 138.5, 128.8, 128.6, 123.6, 108.0, 44.0, 30.5, 27.4; MS: HRMS-ESI-POS: Calcd. for C_26_H_30_N_4_O *m/z* 207.1209 (M^+^+2)/2, found *m/z* 207.1203; Anal. calc. for C_26_H_28_N_4_O-2HCl-2.75H_2_O: C, 58.37; H, 6.68; N, 10.47; Found: C, 58.45; H, 6.54; N, 10.63.

**3,3'-(thiophene-2,5-diylbis(4,1-phenylene)) dipropanimidamide dihydrochloride (DB 1063)**

The dinitrile, 3,3'-(thiophene-2,5-diylbis(4,1-phenylene))dipropanenitrile, was prepared as described for DB1061 yielding a yellow solid 0.77 g (75%); mp 124-6^0^C dec.;^1^H NMR (CDCl_3_): 7.62 (d, 4H, J= 8.0 Hz), 7.29 (d, 4H, J= 8.0 Hz),7.28 (s, 2H), 3.0 (t, 4H, J= 7.2 Hz), 2.66 (t, 4H, J= 7.2 Hz); ^13^C NMR (CDCl_3_): 143.3, 137.5, 133.5, 129.1, 126.2, 124.2, 119.2, 31.4, 19.4; MS: HRMS-ESI-POS: Calc. for C_22_H_18_N_2_SNa *m/z* 365.1088 (M^+^+Na), found *m/z* 365.1089.

Similarly following the DB 1061 procedure the diamidine dihydrochloride was obtained as a yellow solid 0.16 g (66%), mp >280^0^C dec;  ^1^H NMR (DMSO-d_6_): 9.19 (brs, 4H), 8.77 (brs, 4H), 7.64 (d, 4H, J= 8.0 Hz), 7.51 (s, 2H), 7.33 (d, 4H, J=804 Hz), 2.99 (t, 4H, J= 8.4 Hz), 2.73 (d, 4H, J= 8.4 Hz); ^13^C NMR (DMSO-d_6_):170.0, 142.2, 138.9, 131.9, 129.1, 125.3, 124.7, 33.2, 31.6; MS: HRMS-ESI-POS: Calcd. for C_22_H_26_N_4_S *m/z* 189.0939 (M^+^+2)/2, found *m/z* 189.0931; Anal. calcd. for C_22_H_24_N_4_S-2HCl-1.5H_2_O: C, 55.45; H, 6.13; N, 11.76; Found: C, 55.52; H, 6.34; N, 11.63.

**2, 5-bis(4-(2-(4,5-dihydro-1H-imidazol-2-yl) ethyl) phenyl) thiophene dihydrochloride (DB 1064)**

Similarly following the procedure for DB1062 the diamidine dihydrochloride was obtained as a yellow solid, 0.17 g (62%); mp >225°C dec.; ^1^H NMR (DMSO-d_6_): 10.19 (s, 4H), 7.63 (d, 4H, J=7.6 Hz), 7.5 (s, 2H), 7.30 (d, 4H, J= 7.6 Hz), 3.78 (s, 8H),2.97 (t, 4H, J= 6.4 Hz), 2.81(t, 4H, J= 6.4 Hz); ^13^C NMR (DMSO-d6): 170.2, 140.2, 138.9, 131.9, 129.1, 125.4, 124.8, 44.1, 30.6, 27.4; MS: HRMS-ESI-POS: Calcd. for C_26_H_29_N_4_S *m/z* 429.2113 (M^+^+1), found *m/z* 429.2109; Anal. calcd. for C_26_H_28_N_4_S-2HCl-3.0H_2_O: C, 56.21; H, 6.53; N, 10.08; Found: C, 56.34; H, 6.61; N, 10.24.

**4-(5-(1-methyl-1H-benzo[d]imidazol-2-yl) furan-2-yl) benzimidamide dihydrochloride (DB 960)**

To a stirred solution of 5-(4-cyanophenyl) furan-2-aldehyde^2^ 1.23 g (0.005 mole), 1-amino-2-*N*-(methylamino) benzene 0.61 g (0.005 mol) in 20 ml dry DMF under N_2_ was added sodium metabisulfite 0.95 (0.005 mol) and the mixture was heated at 130°C for 12 h (tlc monitored). The solvent was removed, the residue was triturated with cold water, separated solid was filtered, washed with water and air dried. The solid was stirred with 1:1 mixture of DCM-ether, filtered and dried in vac at 70°C for 4 h to give the nitrile, 4-(5-(1-methyl-1H-benzo[d]imidazol-2-yl)furan-2-yl)benzonitrile, as a yellow brown solid, 1.1 g (72%), mp >290°C dec ; ^1^H NMR (DMSO-d_6_): 8.03 (d, 2H, J= 8.4 Hz), 7.92 (d, 2H, J= 8.4 Hz), 7.69-7.63 (m, 2H), 7.46 (d, 1H, J= 3.6 Hz), 7.40 (d, 1H, J= 3.6 Hz), 7.34-7.23 (m, 2H), 4.13 (s, 3H); ^13^C NMR (DMSO-d_6_): 152.4, 145.9, 143.2, 142.4, 136.0, 133.2, 132.9, 124.2, 122.7, 122.2, 118.9, 118.5, 114.7, 111.1, 110.2, 109.9, 31.4; MS: HRMS-ESI-POS: Calcd. for C_19_H_14_N_3_O *m/z* 300.1136 (M^+^+1), found *m/z* 300.1132.

The amidine hydrochloride was obtained as yellow solid (Method B) 0.3 g (78%); mp >300°C dec; ^1^H NMR (DMSO-d_6_): 8.09 (d, 2H, J= 8.7 Hz), 7.90 (d, 2H, J= 8.7 Hz), 7.78-7.70 (m, 2H), 7.61 (d, 1H, J= 3.9 Hz), 7.48-7.42 (m, 2H), 7.45 (d, 1H, J= 3.9 Hz), 4.13 (s, 1H); ^13^C NMR (DMSO-d_6_): 165.5, 155.6, 141.5, 141.3, 135.3, 134.8, 133.9, 129.5, 128.0, 126.0, 125.8, 125.5, 119.6, 116.4, 112.4, 112.0, 32.9; MS: HRMS-ESI-POS: Calcd. for C_19_H_18_N_4_O *m/z* 159.0740 (M^+^+2)/2, found *m/z* 159.0733; Anal. calcd. for C_19_H_16_N_4_O-2HCl-1H_2_O: C, 56.02; H, 4.94; N, 13.76; Found: C, 56.18; H, 4.91; N, 13.51.

**References**

1. Depauw, S.; Lambert, M.; Jambon, S.; Paul, A.; Peixoto, P.; Nhili, R.; Marongiu, L.; Figeac, M.; Dassi, C.; Paul-Constant, C.; Billoré, B.; Kumar, A.; Farahat, A.; Ismail, M.; Mineva, E.; Sweat , D. ; Stephens, C.; Boykin, D.; Wilson, W.; David-Cordonnier, M-H. Heterocyclic Diamidines DNA ligands as HOXA9 Transcription Factor Inhibitors: Design, Molecular Evaluation and Cellular Consequences in HOXA9-Dependant Leukemia Cell Model. *J Med Chem* **62,** 1306-1329 (2019).
2. Mitsch, A., Wissner, P., Silber, K., Hacebel, P., Sattler, I., Klebe, G., Schlitzer, M. Non-thiol farnesyltransferase inhibitors: *N*-(4-tolylacetylamino-3-benzoylphenyl)-3-arylfurylacrylic acid amides. Bioorg. & Med. Chem. **12**, 4585-4600 (2004).
