# Supplementary material for: Positively selected modifications in the pore of TbAQP2 allow pentamidine to enter Trypanosoma brucei
Source: eLife. 2020 Aug 11;9:e56416. doi: 10.7554/eLife.56416 (PMC7473772; doi:10.7554/eLife.56416)
Supplement: Supplementary file 4. [file elife-56416-supp4.docx]

| Mutation | Primer | Sequence (altered base(s) underlined) | Generated Plasmid | Original Template |
| --- | --- | --- | --- | --- |
| S131P/S263A | S131P: HDK1062 | CTCCGGTGGCCATCTCAACCCTGCCGTCACCGTTGGCAA | pHDK166 | pRPa^GFP-AQP2^ |
|  | S131P: HDK1063 | TTGCCAACGGTGACGGCAGGGTTGAGATGGCCACCGGAG |  |  |
|  | S263A: HDK1064 | TCTCCCCTTGCGATGAATCCCGCACTTGATTTCGGTCCCAGGG |  |  |
|  | S263A: HDK1065 | CCCTGGGACCGAAATCAAGTGCGGGATTCATCGCAAGGGGAGA |  |  |
| I110W | HDK607 | CTCGGTCTTACGATTGGTTGGGGTGTGGCTGTCACGATG | pHDK84 | pRPa^GFP-AQP2^ |
|  | HDK608 | CATCGTGACAGCCACACCCCAACCAATCGTAAGACCGAG |  |  |
| L264R | HDK609 | TCTCCCCTTGCGATGAATCCCTCACGTGATTTCGGTCCCAGGGTCTTC | pHDK167 | pRPa^GFP-AQP2^ |
|  | HDK610 | GAAGACCCTGGGACCGAAATCACGTGAGGGATTCATCGCAAGGGGAGA |  |  |
| I110W/L264R | L264R: HDK609 | TCTCCCCTTGCGATGAATCCCTCACGTGATTTCGGTCCCAGGGTCTTC | pHDK78 | pHDK84 |
|  | L264R: HDK610 | GAAGACCCTGGGACCGAAATCACGTGAGGGATTCATCGCAAGGGGAGA |  |  |
| L258Y | HDK1109 | CAACTTCGGCTTAGCGTCTCCCTATGCGATGAATCCCTCACTTGAT | pHDK168 | pRPa^GFP-AQP2^ |
|  | HDK1110 | ATCAAGTGAGGGATTCATCGCATAGGGAGACGCTAAGCCGAAGTTG |  |  |
| I190T | HDK1056 | GCCTTCGGTGAAAAGGGGACTGCGTGGGTGTTTGCCATG | pHDK163 | pRPa^GFP-AQP2^ |
|  | HDK1057 | CATGGCAAACACCCACGCAGTCCCCTTTTCACCGAAGGC |  |  |
| W192G | HDK1058 | CGGTGAAAAGGGGATTGCGGGGGTGTTTGCCATGTACCC | pHDK164 | pRPa^GFP-AQP2^ |
|  | HDK1059 | GGGTACATGGCAAACACCCCCGCAATCCCCTTTTCACCG |  |  |
| I190T/W192G | HDK1060 | GCCTTCGGTGAAAAGGGGACTGCGGGGGTGTTTGCCATGTACCC | pHDK165 | pHDK163 |
|  | HDK1061 | GGGTACATGGCAAACACCCCCGCAGTCCCCTTTTCACCGAAGGC |  |  |
| L84W | HDK1276 | AAACTTCGTCTGGATATATATCGCTAAGGG | pHDK210 | pRPa^GFP-AQP2^ |
|  | HDK1277 | CCCAGAAATTCAGCCACG |  |  |
| L118W | HDK1274 | CACCGCAGTGTGGCTGCTCTGTG | pHDK208 | pRPa^GFP-AQP2^ |
|  | HDK1275 | GAAATGAGTTCAGCAAAAATTG |  |  |
| L218W | HDK1272 | CACGATGGCTTGGTATGTTTCACTG | pHDK209 | pRPa^GFP-AQP2^ |
|  | HDK1273 | ACAGCCACACCAATACCA |  |  |
| L84W/L118W | HDK1276 | AAACTTCGTCTGGATATATATCGCTAAGGG | pHDK227 | pHDK208 |
|  | HDK1277 | CCCAGAAATTCAGCCACG |  |  |
| L84M | HDK1364 | AAACTTCGTCATGATATATATCGCTAAGG | pHDK234 | pHDK210 |
|  | HDK1367 | GAAATGAGTTCAGCAAAAATTGGATAAAATATAC |  |  |
| L118M | HDK1365 | CACGATGGCTATGTATGTTTCAC | pHDK235 | pHDK208 |
|  | HDK1367 | GAAATGAGTTCAGCAAAAATTGGATAAAATATAC |  |  |
| L218M | HDK1366 | CACCGCAGTGATGCTGCTCTGTG | pHDK236 | pHDK209 |
|  | HDK1367 | GAAATGAGTTCAGCAAAAATTGGATAAAATATAC |  |  |

**Supplementary File 4. List of primers used in the construction of mutations in TbAQP2.**
