# Supplementary material for: Positively selected modifications in the pore of TbAQP2 allow pentamidine to enter Trypanosoma brucei
Source: eLife. 2020 Aug 11;9:e56416. doi: 10.7554/eLife.56416 (PMC7473772; doi:10.7554/eLife.56416)
Supplement: Supplementary file 5. [file elife-56416-supp5.docx]

**Supplementary File 5. Primers used for mutations in TbAQP3.**

| Mutations | Primer | Sequence (altered base(s) underlined) | Plasmid | Template |
| --- | --- | --- | --- | --- |
| W102I/R256L | W102I: HDK511 | CTCAGCATTACGCTTGGTATCGGCATTGCCGTCACGATG | pHDK71 | pRPa^GFP-AQP3^ |
|  | W102I: HDK512 | CATCGTGACGGCAATGCCGATACCAAGCGTAATGCTGAG |  |  |
|  | R256L: HDK513 | TACGCAATAAATCCGGCTCTTGACTTCGGTCCCAGGGTC |  |  |
|  | R256L: HDK514 | GACCCTGGGACCGAAGTCAAGAGCCGGATTTATTGCGTA |  |  |
| W102I/R256L/Y250L | Y250L HDK795 | CATCGGTTACTCAACGGGTCTCGCAATAAATCCGGCTCTT | pHDK121 | pHDK71 |
|  | Y250L HDK796 | AAGAGCCGGATTTATTGCGAGACCCGTTGAGTAACCGATG |  |  |
